# Synthesis, crystal structure and Hirshfeld surface analysis of bis­[2-amino-5-(ethyl­sulfan­yl)-1,3,4-thia­diazol-3-ium] bis­(perchlorato-κ*O*)bis­(picolinato-κ^2^*N*,*O*)copper(II)

**DOI:** 10.1107/S2056989025004992

**Published:** 2025-06-12

**Authors:** Gulnaz Khojabaeva, Batirbay Torambetov, Rajesh G. Gonnade, Zamira Uzakbergenova, Abdusamat Rasulov, Shakhnoza Kadirova

**Affiliations:** a National University of Uzbekistan named after Mirzo Ulugbek 4 University St Tashkent 100174 Uzbekistan; b Physical and Material Chemistry Division CSIR-National Chemical Laboratory,Pune 411008 India; c Academy of Scientific and Innovative Research (AcSIR) Sector 19 Kamla Nehru Nagar Ghaziabad Uttar Pradesh 201002 India; d Karakalpak State University, 1 Ch. Abdirov St. Nukus, 230112, Uzbekistan; e Termez University of Economics and Service, 41B Farovon St., Termiz, 190111, Uzbekistan; University of Neuchâtel, Switzerland

**Keywords:** crystal structure, copper(II) complex, picolinate ligand, thia­diazole cation, Hirshfeld surface analysis, hydrogen bonding, supra­molecular inter­actions, Jahn–Teller distortion

## Abstract

In the title coordination complex, (C_4_H_8_N_3_S_2_)_2_[Cu(C_6_H_4_NO_2_)_2_(ClO_4_)_2_], the Cu^II^ center exhibits a distorted octa­hedral geometry with an equatorial plane formed by two bidentate picolinato ligands and axial coordination from two perchlorate anions, consistent with a Jahn–Teller distortion. The crystal structure is further consolidated by a network of hydrogen bonds involving the thia­diazole-based cations, which reside in the outer coordination sphere in a unique protonated state. Hirshfeld surface analysis reveals that O⋯H/H⋯O contacts dominate the inter­molecular inter­actions, contributing significantly to the crystal packing.

## Abstract 2

The title copper(II) complex exhibits a distorted octa­hedral geometry in which the Cu^II^ cation is coordinated by the bidentate picolinate and monodentate perchlorate ligands. The crystal structure features a unique outer-sphere protonated thia­diazole cation inter­acting *via* hydrogen bonds. Hirshfeld surface analysis underscores the dominant role of O⋯H/H⋯O inter­actions in the crystal packing.

## 1. Chemical context

The coordination chemistry of transition-metal complexes with heterocyclic ligands has attracted substantial attention because of their diverse applications in catalysis, medicinal chemistry, and materials science. Among such ligands, picolinate anions and 1,3,4-thia­diazole derivatives are especially prominent due to their strong metal-binding abilities and biological relevance. Picolinic acid and its derivatives are well-known for their bidentate coordination modes, often employed in the design of metal–organic complexes to modulate metal center reactivity and bioactivity (Lavrenova *et al.*, 2023 ▸). Similarly, 1,3,4-thia­diazole derivatives are recognized for their wide spectrum of biological properties, including anti­microbial, anti­cancer, and anti­diabetic activities (Hu *et al.*, 2014 ▸; Gond *et al.*, 2022 ▸; Dani *et al.*, 2015 ▸). These heterocycles, containing electron-donating nitro­gen and sulfur atoms, readily coordinate with transition metals, thereby influencing the physicochemical and pharmacological properties of the resulting complexes (Kadirova *et al.*, 2022 ▸; Atashov *et al.*, 2024 ▸). Transition metals such as zinc(II), nickel(II), and cobalt(II) have been extensively studied in coordination with thia­diazole-based ligands. Zinc(II) complexes have shown promise in drug delivery and enzyme mimicry (Shen *et al.*, 2004 ▸), while nickel(II) and cobalt(II) derivatives exhibit significant catalytic activity and electrochemical potential (Song *et al.*, 2012 ▸; Ishankhodzhaeva *et al.*, 2001 ▸; Ma *et al.*, 2018 ▸; Nuralieva *et al.*, 2025 ▸). These findings underscore the versatility of thia­diazole coordination chemistry across various metal centers. Among transition metals, copper(II) occupies a unique position due to its redox flexibility and biological significance. Copper(II) complexes incorporating 1,3,4-thia­diazole ligands have been investigated extensively for their enhanced anti­microbial and anti­cancer properties compared to the free ligands (Karcz *et al.*, 2020 ▸; Heidari *et al.*, 2020 ▸). The synergistic effect between the copper center and heterocyclic ligand often leads to improved biological efficacy, making them attractive scaffolds for drug development (Gurbanov *et al.*, 2023 ▸; Camí *et al.*, 2005 ▸).
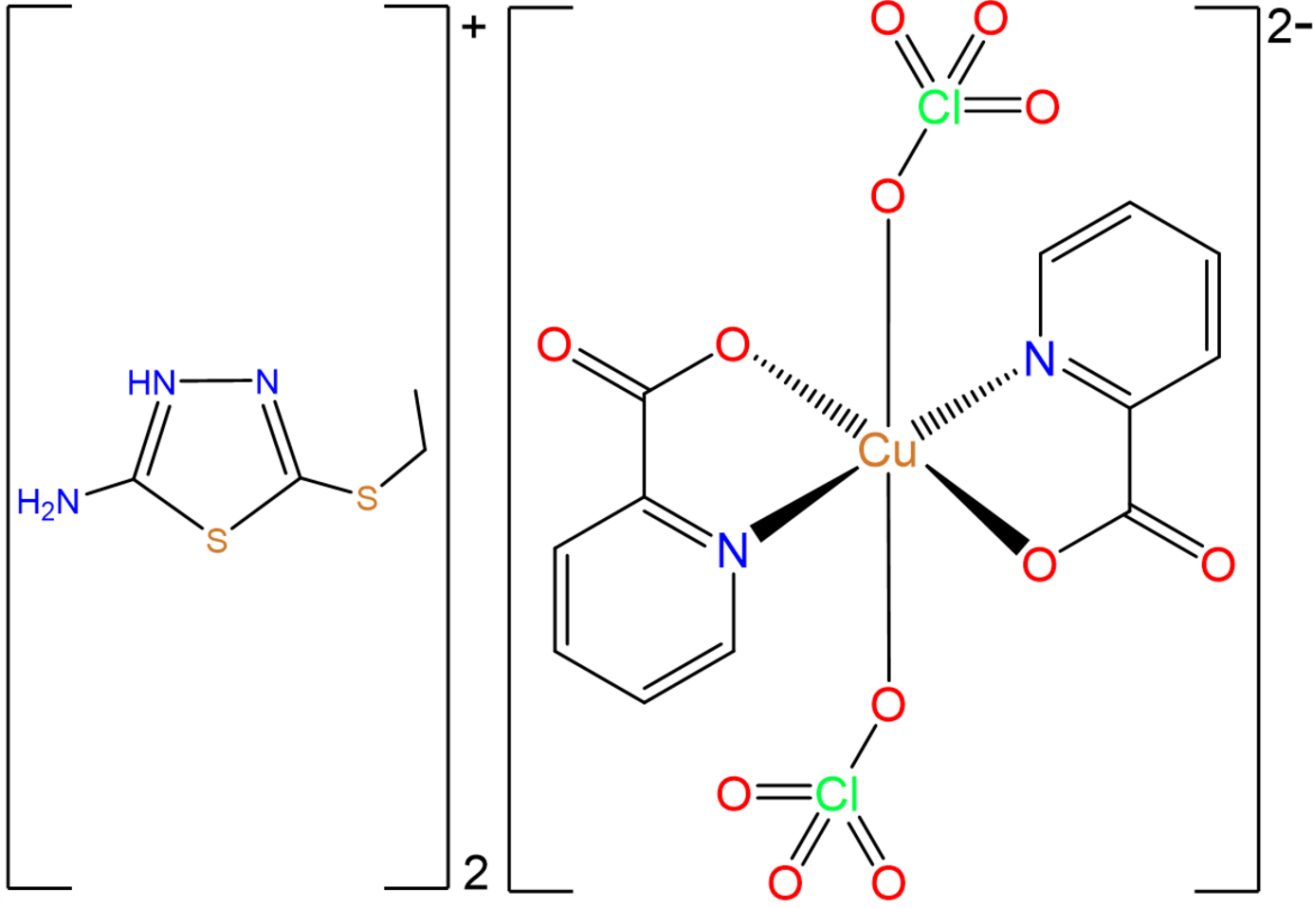


In this context, we present the synthesis, structural characterization, and Hirshfeld surface analysis of the complex (H*L*)_2_[Cu(Pic)_2_(ClO_4_)_2_], where Pic is the picolinate anion and H*L* is the 2-amino-5-ethyl­thio-1,3,4-thia­diazol-3-ium cation. The structural investigation, carried out using single-crystal X-ray diffraction (SC-XRD), aimed to elucidate the coordination environment, inter­molecular inter­actions, and potential implications for biological activity.

## 2. Structural commentary

**Figure 1:**
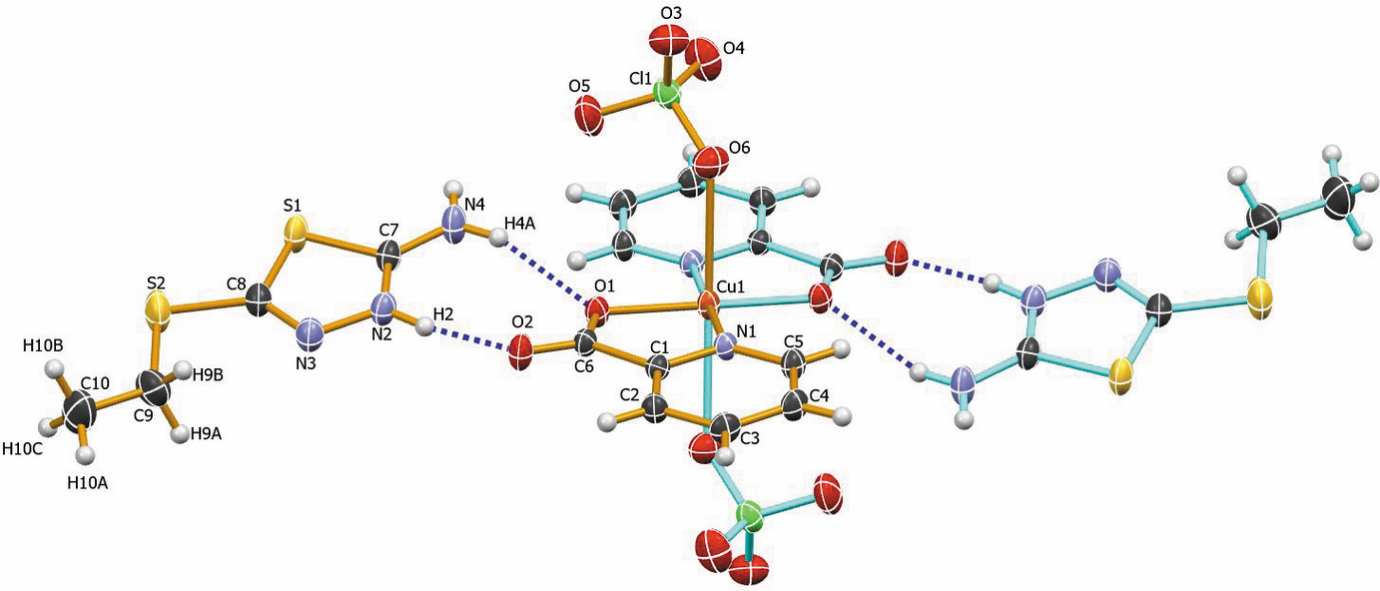
Mol­ecular structure of the title copper(II) complex showing the coordination environment around the Cu^II^ center. Displacement ellipsoids are drawn at the 50% probability level. Hydrogen bonds are indicated by blue dashed lines.

Single-crystal X-ray diffraction analysis reveals that (H*L*)_2_[Cu(Pic)_2_(ClO_4_)_2_] crystallizes in the triclinic *P*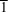 space group. The asymmetric unit consists of half of a Cu^II^ cation, one picolinate anion, one perchlorate anion and one protonated 2-amino-5-ethyl­thio-1,3,4-thia­diazole (H*L*) ligand. The Cu^II^ cation is positioned on an inversion center and adopts an axially elongated octa­hedral coordination geometry (Fig. 1 ▸). The equatorial plane consists of two nitro­gen atoms (N1) and two carboxyl­ate oxygen atoms from symmetry-related picolinate ligands, forming a nearly planar Cu(pic)_2_ moiety. The axial sites are occupied by two perchlorate anions, each weakly coordinating to the Cu^II^ center. The elongated Cu1—O6 distance of 2.532 (2) Å exceeds the typical Cu—O coordination bond range (1.8–2.0 Å) (Veidis *et al.*, 1969 ▸), consistent with a Jahn–Teller distortion along the O6–Cu–O6 axis. The Cu(Pic)_2_ moiety is nearly planar, with the root-mean-square (r.m.s.) deviations of the oxygen atoms being 0.039 Å for O1 and 0.073 Å for O2, indicating a high degree of planarity in this coordination environment. The geometry and bond lengths of the coordinated picolinato ligands are characteristic of a deprotonated carboxyl­ate group (Fábry, 2018 ▸), with the coordinated C—O bond length [1.281 (3) Å] being slightly longer than the C=O bond [1.231 (3) Å].

## 3. Supra­molecular features

**Figure 2:**
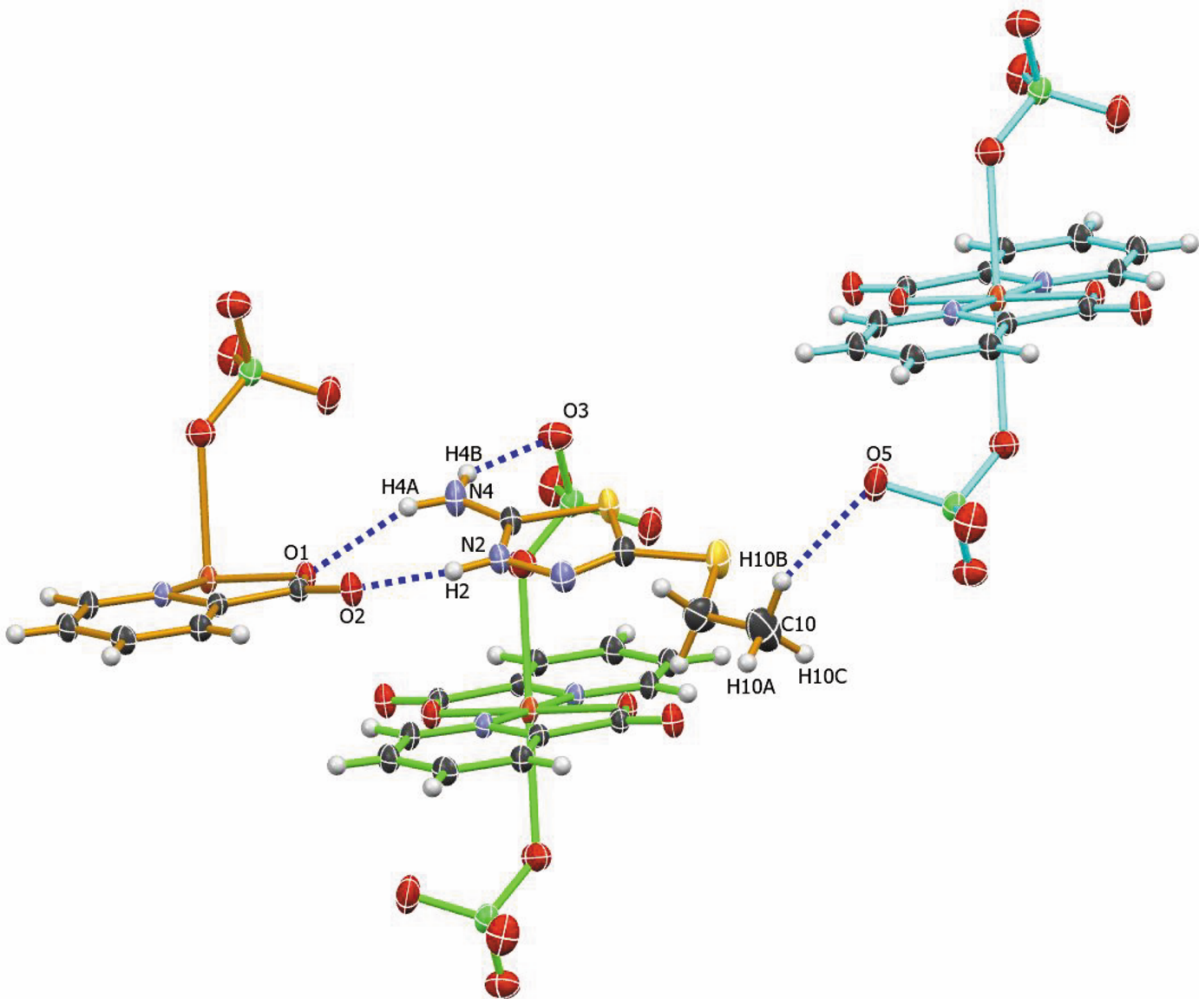
Packing diagram of the title complex showing inter­molecular hydrogen bonding (blue dashed lines) between the thia­diazole cation and coordinated perchlorate anions, contributing to the supra­molecular architecture. Different mol­ecular fragments are color-coded for clarity. Displacement ellipsoids are drawn at the 50% probability level.

In the extended structure, the [Cu(pic)_2_(ClO_4_)_2_]^2−^ units are assembled into a supra­molecular network through hydrogen-bonding inter­actions with the H*L* protonated thia­diazole cations (Fig. 2 ▸, Table 1 ▸). The crystal packing is governed by two distinct types of hydrogen bonds: inter­molecular and intra­molecular. Inter­molecular hydrogen bonds are observed between the O3 atom of the perchlorate anion and the amine hydrogen atom of the thia­diazole cation (O3⋯H4*B* = 2.21 Å), as well as between the O5 atom of the perchlorate anion and the hydrogen atom of the methyl group of the thia­diazole cation (O5⋯H10*B* = 2.32 Å). Intra­molecular hydrogen bonds occur between the protonated H2*A* atom and atom H4*A* of the thiadiazole ring with carboxyl­ate atoms O2 and O1 in the pic ligands (H2*A*⋯O2 = 1.80 Å, H4*A*⋯O1 = 2.14 Å), forming eight-membered ring motifs with an 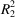
(8) graph set (Bernstein *et al.*, 1995 ▸). These non-covalent inter­actions play a crucial role in the cohesion of the three-dimensional crystal architecture.

## 4. Hirshfeld Surface Analysis

**Figure 3:**
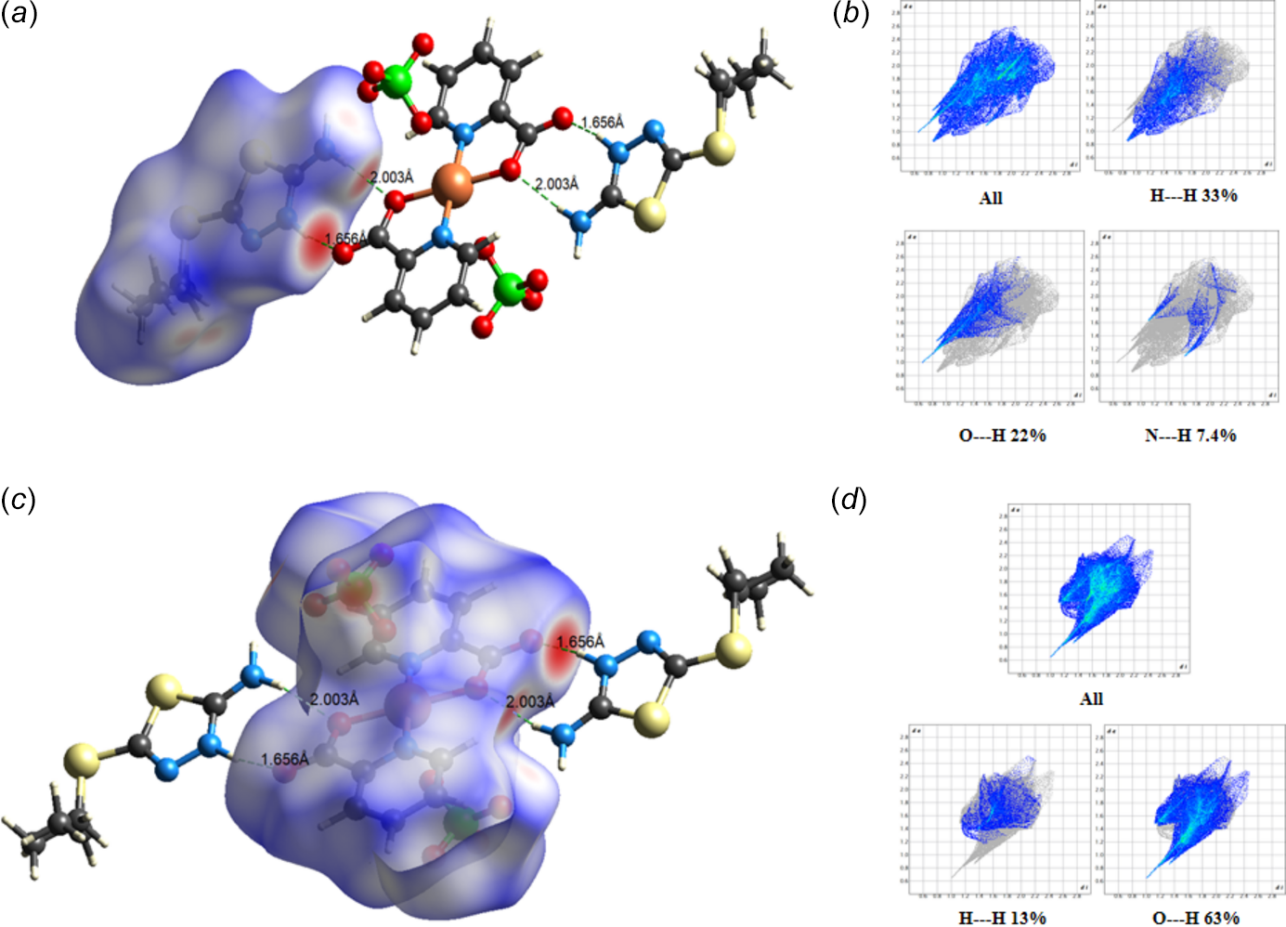
Different views of the Hirshfeld surfaces (*a*) thia­diazole (H*L*) cation, (*b*) two-dimensional fingerprint plots of the H*L* cation, (*c*) picolinate/perchlorate anions and (*d*) two-dimensional fingerprint plots of the sulfate anion and picolinate/perchlorate anions.

Hirshfeld surface (HS) analysis was performed and two-dimensional fingerprint (FP) plots were generated using *CrystalExplorer 21.5* (Spackman *et al.*, 2021 ▸) to systematically examine the inter­molecular inter­actions governing the crystal packing. In the HS diagram, contacts with inter­atomic distances equal to the sum of van der Waals radii are represented in white, while those shorter and longer than this threshold appear in red and blue, respectively. The FP plots provide a qu­anti­tative assessment of the relative contributions of distinct inter­molecular inter­actions. These plots were generated based on the distances *d*_e_ (the distance from the HS to the nearest external atom) and *d*_i_ (the distance from the HS to the nearest inter­nal atom). Prior to HS calculations, O—H bond lengths were standardized to neutron diffraction values (0.983 Å) to ensure computational accuracy. HS analysis was conducted separately for the cationic and anionic motifs to delineate their individual contributions to the overall crystal packing. The inter­molecular O⋯H/H⋯O inter­actions were found to be predominant, contributing 22% to the HS area in the thia­diazole (H*L*) cation and 63% in the picolinate/perchlorate anions. Additionally, H⋯H contacts accounted 33% for thia­diazole (H*L*) cation and 13% for picolinate/perchlorate anions. The FP plots (Fig. 3 ▸) further elucidate these contributions. The HS analysis unequivocally reveals that the title compound is primarily consolidated by a network of O—H⋯O hydrogen bonds, which facilitate the formation of an extended three-dimensional supra­molecular architecture in the solid state.

## 5. Database survey

A survey conducted using ConQuest software (CSD, Version 5.46, November 2024; Groom *et al.*, 2016 ▸) within the Cambridge Structural Database revealed 311 metal complex crystal structures in which two picolinate anions are coordinated bidentately to a metal center. Among these, only two structures [NEBQAP (Csonka *et al.*, 2018 ▸) and TAFNAQ (Guo *et al.*, 2003 ▸)] feature a perchlorate anion directly coordinated to a copper(II) atom. Additionally, 22 organometallic crystal structures containing 2-amino-5-mercapto-1,3,4-thia­diazole derivatives have been reported, and in all cases, the thia­diazole ligand binds to the metal through the nitro­gen atom at the 3-position [CADMIH (Heidari *et al.*, 2020 ▸); CEDSEM (Slyvka, 2017*a* ▸); ESIBUG (Slyvka *et al.*, 2021 ▸); FEXPUX (Zou *et al.*, 2023 ▸); GAKMOX (Mu *et al.*, 2016 ▸); HAJLUC, HAJMAJ and HAJMIR (Ardan *et al.*, 2017 ▸); HONDOG (Torambetov *et al.*, 2019 ▸); JIYTEU (Gurbanov *et al.*, 2023 ▸); JIZKEK and JIZKEK01 (Soudani *et al.*, 2014 ▸); JOJLUT (Kadirova *et al.*, 2022 ▸); LIFCEK (Hu *et al.*., 2012*a* ▸); LOKYIX (Atashov *et al.*, 2024 ▸); ODAPOC (Slyvka *et al.*, 2022 ▸); TEGWIN (Hu *et al.*, 2012*b>* ▸); XIGWEQ, XIGWIU, XIGWOA and XIGWUG (Camí *et al.*, 2005 ▸); YEBNAX (Slyvka, 2017*b* ▸). Remarkably, no crystal structures have been reported in which the 1,3,4-thia­diazole-based ligand exists in a protonated form and is located in the outer coordination sphere of the complex as a cation. These observations underscore the novelty of the structural motif reported in the present work.

## 6. Synthesis and crystallization

Cu(ClO_4_)_2_·6H_2_O (0.185 g, 0.5 mmol) and picolinic acid (0.123 g, 1 mmol) were each dissolved separately in methanol (3 mL). The two solutions were then mixed and stirred at 323 K for 1 h. A solution of *L* (2-amino-5-ethyl­thio-1,3,4-thia­diazole) was prepared by dissolving *L* (0.161 g, 1 mmol) in 3 mL of methanol. This solution was added dropwise to the previously prepared copper(II) perchlorate–picolinic acid solution, resulting in the formation of a blue solution. Upon continuous stirring at 323 K for 4 h, the color of the reaction mixture gradually changed to green. The solution was then filtered and left to crystallize. Single crystals of the title complex, suitable for X-ray diffraction analysis, were obtained by slow evaporation of the filtrate over a period of 7 days.

## 7. Refinement

Crystal data, data collection and structure refinement details are summarized in Table 2 ▸. H atoms were positioned geometrically (N—H = 0.86 Å, C—H = 0.93–0.97 Å) and refined as riding with *U*_iso_(H) = 1.2*U*_eq_(N, C) or 1.5*U*_eq_(C-meth­yl). The ethyl group of the thia­diazole mol­ecule is disordered over two positions, C9 and C10, with equal occupancies.

## Supplementary Material

Crystal structure: contains datablock(s) I. DOI: 10.1107/S2056989025004992/tx2099sup1.cif

Structure factors: contains datablock(s) I. DOI: 10.1107/S2056989025004992/tx2099Isup2.hkl

CCDC reference: 2455854

Additional supporting information:  crystallographic information; 3D view; checkCIF report

## Figures and Tables

**Table 1:** Hydrogen-bond geometry (Å, °)

*D*—H⋯*A*	*D*—H	H⋯*A*	*D*⋯*A*	*D*—H⋯*A*
N4—H4*A*⋯O1	0.86	2.14	2.940 (3)	156
N4—H4*A*⋯O5	0.86	2.59	2.951 (3)	106
N4—H4*B*⋯O3^i^	0.86	2.21	2.981 (3)	150
N4—H4*B*⋯O6^i^	0.86	2.57	3.005 (3)	113
N2—H2⋯O2	0.86	1.81	2.656 (3)	170

Symmetry code: (i) 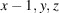.

**Table 2:** Experimental details

Crystal data	
Chemical formula	(C_4_H_8_N_3_S_2_)_2_[Cu(C_6_H_4_NO_2_)_2_(ClO_4_)_2_]
*M*_r_	828.13
Crystal system, space group	Triclinic, *P* 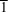
Temperature (K)	299
*a*, *b*, *c* (Å)	6.3100 (2), 9.8179 (2), 13.3493 (4)
α, β, γ (°)	90.522 (1), 98.818 (1), 99.478 (1)
*V* (Å^3^)	805.57 (4)
*Z*	1
Radiation type	Mo *K*α
μ (mm^−1^)	1.17
Crystal size (mm)	0.11 × 0.1 × 0.06

Data collection	
Diffractometer	Bruker D8 VENTURE Kappa Duo PHOTON II CPAD
Absorption correction	Multi-scan (*SADABS*; Krause *et al.*, 2015 ▸)
*T*_min_, *T*_max_	0.633, 0.745
No. of measured, independent and observed [*I* > 2σ(*I*)] reflections	28717, 3315, 2937
*R*_int_	0.057
(sin θ/λ)_max_ (Å^−1^)	0.628

Refinement	
*R*[*F*^2^ > 2σ(*F*^2^)], *wR*(*F*^2^), *S*	0.035, 0.099, 1.03
No. of reflections	3315
No. of parameters	233
No. of restraints	24
H-atom treatment	H-atom parameters constrained
Δρ_max_, Δρ_min_ (e Å^−3^)	0.40, −0.30

Computer programs: *APEX3* and *SAINT* (Bruker, 2016 ▸), *SHELXT2018/2* (Sheldrick, 2015*a* ▸), *SHELXL2016/6* (Sheldrick, 2015*b* ▸) and *OLEX2* (Dolomanov *et al.*, 2009 ▸).

## supplementary crystallographic information

### Bis[2-amino-5-(ethylsulfanyl)-1,3,4-thiadiazol-3-ium] bis(perchlorato-κO)bis(picolinato-κ2N,O)copper(II) Crystal data

**Table:**

(C_4_H_8_N_3_S_2_)_2_[Cu(C_6_H_4_NO_2_)_2_(ClO_4_)_2_]	*Z* = 1
*M**_r_* = 828.13	*F*(000) = 420
Triclinic, *P*1	*D*_x_ = 1.707 Mg m^−^^3^
*a* = 6.3100 (2) Å	Mo *K*α radiation, λ = 0.71073 Å
*b* = 9.8179 (2) Å	Cell parameters from 9876 reflections
*c* = 13.3493 (4) Å	θ = 2.6–26.4°
α = 90.522 (1)°	µ = 1.17 mm^−^^1^
β = 98.818 (1)°	*T* = 299 K
γ = 99.478 (1)°	Block, green
*V* = 805.57 (4) Å^3^	0.11 × 0.1 × 0.06 mm

### Bis[2-amino-5-(ethylsulfanyl)-1,3,4-thiadiazol-3-ium] bis(perchlorato-κO)bis(picolinato-κ2N,O)copper(II) Data collection

**Table:**

Bruker D8 VENTURE Kappa Duo PHOTON II CPAD diffractometer	2937 reflections with *I* > 2σ(*I*)
Detector resolution: 7.39 pixels mm^-1^	*R*_int_ = 0.057
φ and ω scans	θ_max_ = 26.5°, θ_min_ = 2.6°
Absorption correction: multi-scan (SADABS; Krause *et al.*, 2015)	*h* = −7→7
*T*_min_ = 0.633, *T*_max_ = 0.745	*k* = −12→12
28717 measured reflections	*l* = −16→16
3315 independent reflections	

### Bis[2-amino-5-(ethylsulfanyl)-1,3,4-thiadiazol-3-ium] bis(perchlorato-κO)bis(picolinato-κ2N,O)copper(II) Refinement

**Table:**

Refinement on *F*^2^	24 restraints
Least-squares matrix: full	Hydrogen site location: inferred from neighbouring sites
*R*[*F*^2^ > 2σ(*F*^2^)] = 0.035	H-atom parameters constrained
*wR*(*F*^2^) = 0.099	*w* = 1/[σ^2^(*F*_o_^2^) + (0.0476*P*)^2^ + 0.4379*P*] where *P* = (*F*_o_^2^ + 2*F*_c_^2^)/3
*S* = 1.03	(Δ/σ)_max_ < 0.001
3315 reflections	Δρ_max_ = 0.40 e Å^−^^3^
233 parameters	Δρ_min_ = −0.30 e Å^−^^3^

### Bis[2-amino-5-(ethylsulfanyl)-1,3,4-thiadiazol-3-ium] bis(perchlorato-κO)bis(picolinato-κ2N,O)copper(II) Special details

**Table:**

Geometry. All esds (except the esd in the dihedral angle between two l.s. planes) are estimated using the full covariance matrix. The cell esds are taken into account individually in the estimation of esds in distances, angles and torsion angles; correlations between esds in cell parameters are only used when they are defined by crystal symmetry. An approximate (isotropic) treatment of cell esds is used for estimating esds involving l.s. planes.

### Bis[2-amino-5-(ethylsulfanyl)-1,3,4-thiadiazol-3-ium] bis(perchlorato-κO)bis(picolinato-κ2N,O)copper(II) Fractional atomic coordinates and isotropic or equivalent isotropic displacement parameters (Å^2^)

**Table:**

	*x*	*y*	*z*	*U*_iso_*/*U*_eq_	Occ. (<1)
Cu1	0.500000	0.500000	0.000000	0.04229 (13)	
S1	−0.22380 (12)	0.45846 (8)	0.36426 (6)	0.0638 (2)	
S2	−0.33608 (16)	0.66980 (10)	0.50029 (7)	0.0859 (3)	
Cl1	0.55824 (9)	0.20197 (6)	0.16167 (5)	0.05452 (17)	
O1	0.3708 (2)	0.55987 (16)	0.11468 (13)	0.0472 (4)	
O2	0.4332 (3)	0.72184 (19)	0.23898 (14)	0.0593 (5)	
O3	0.7223 (4)	0.1767 (2)	0.24227 (18)	0.0776 (6)	
O4	0.4945 (4)	0.0857 (2)	0.0948 (2)	0.0889 (7)	
O5	0.3737 (4)	0.2359 (3)	0.1990 (2)	0.0906 (7)	
O6	0.6536 (3)	0.3183 (2)	0.10864 (18)	0.0729 (6)	
N3	−0.0062 (4)	0.7053 (2)	0.39137 (18)	0.0620 (6)	
N4	0.0747 (4)	0.4205 (3)	0.24606 (18)	0.0657 (6)	
H4A	0.189153	0.449554	0.219641	0.079*	
H4B	0.008829	0.336685	0.234388	0.079*	
N1	0.7410 (3)	0.65204 (18)	0.05284 (14)	0.0404 (4)	
N2	0.0878 (3)	0.6329 (2)	0.32667 (16)	0.0558 (5)	
H2	0.200239	0.671103	0.301887	0.067*	
C1	0.6886 (3)	0.7281 (2)	0.12672 (16)	0.0400 (4)	
C2	0.8134 (4)	0.8502 (2)	0.16490 (19)	0.0488 (5)	
H2A	0.772326	0.900672	0.215821	0.059*	
C3	1.0027 (4)	0.8970 (3)	0.1258 (2)	0.0541 (6)	
H3	1.089572	0.980444	0.149272	0.065*	
C4	1.0600 (4)	0.8186 (3)	0.0522 (2)	0.0521 (6)	
H4	1.188109	0.847296	0.026328	0.063*	
C5	0.9263 (3)	0.6967 (2)	0.01687 (18)	0.0471 (5)	
H5	0.965821	0.643983	−0.033273	0.057*	
C6	0.4818 (4)	0.6673 (2)	0.16415 (17)	0.0434 (5)	
C7	0.0006 (4)	0.5040 (3)	0.30408 (18)	0.0503 (5)	
C8	−0.1698 (4)	0.6261 (3)	0.4178 (2)	0.0572 (6)	
C9	−0.218 (3)	0.8510 (10)	0.5248 (9)	0.089 (3)	0.5
H9B	−0.063195	0.856523	0.546730	0.106*	0.5
H9A	−0.236435	0.897343	0.460917	0.106*	0.5
C10	−0.299 (2)	0.9253 (9)	0.5957 (8)	0.124 (4)	0.5
H10A	−0.232139	1.020680	0.596917	0.186*	0.5
H10B	−0.265597	0.888642	0.661633	0.186*	0.5
H10C	−0.453377	0.917710	0.577437	0.186*	0.5
C9A	−0.297 (3)	0.869 (2)	0.4976 (17)	0.180 (9)	0.5
H9AA	−0.241457	0.906202	0.437962	0.215*	0.5
H9AB	−0.424855	0.906473	0.508259	0.215*	0.5
C10A	−0.135 (3)	0.878 (2)	0.5860 (14)	0.169 (6)	0.5

### Bis[2-amino-5-(ethylsulfanyl)-1,3,4-thiadiazol-3-ium] bis(perchlorato-κO)bis(picolinato-κ2N,O)copper(II) Atomic displacement parameters (Å^2^)

**Table:**

	*U*^11^	*U*^22^	*U*^33^	*U*^12^	*U*^13^	*U*^23^
Cu1	0.0311 (2)	0.0428 (2)	0.0522 (2)	−0.00334 (14)	0.01479 (15)	−0.00666 (16)
S1	0.0555 (4)	0.0684 (4)	0.0696 (4)	−0.0064 (3)	0.0344 (3)	−0.0101 (3)
S2	0.0874 (6)	0.0956 (6)	0.0854 (6)	0.0158 (5)	0.0478 (5)	−0.0145 (5)
Cl1	0.0440 (3)	0.0499 (3)	0.0709 (4)	0.0048 (2)	0.0168 (3)	−0.0048 (3)
O1	0.0375 (8)	0.0445 (8)	0.0598 (9)	−0.0041 (6)	0.0203 (7)	−0.0047 (7)
O2	0.0535 (10)	0.0640 (11)	0.0612 (10)	−0.0048 (8)	0.0277 (8)	−0.0129 (8)
O3	0.0809 (14)	0.0635 (12)	0.0845 (14)	0.0103 (10)	0.0026 (11)	0.0113 (10)
O4	0.0774 (15)	0.0731 (14)	0.1099 (18)	−0.0060 (11)	0.0173 (13)	−0.0357 (13)
O5	0.0708 (14)	0.0922 (16)	0.123 (2)	0.0205 (12)	0.0520 (14)	0.0009 (14)
O6	0.0512 (11)	0.0727 (12)	0.0931 (15)	0.0042 (9)	0.0114 (10)	0.0221 (11)
N3	0.0627 (14)	0.0594 (13)	0.0663 (14)	0.0038 (10)	0.0249 (11)	−0.0047 (10)
N4	0.0539 (13)	0.0712 (14)	0.0735 (15)	−0.0049 (11)	0.0320 (11)	−0.0152 (12)
N1	0.0308 (8)	0.0420 (9)	0.0477 (10)	0.0006 (7)	0.0104 (7)	−0.0008 (7)
N2	0.0483 (11)	0.0608 (12)	0.0595 (12)	0.0003 (9)	0.0222 (10)	0.0002 (10)
C1	0.0336 (10)	0.0408 (10)	0.0459 (11)	0.0036 (8)	0.0104 (8)	0.0037 (9)
C2	0.0479 (13)	0.0439 (12)	0.0541 (13)	0.0015 (9)	0.0137 (10)	−0.0053 (10)
C3	0.0463 (13)	0.0447 (12)	0.0662 (15)	−0.0089 (10)	0.0115 (11)	−0.0027 (11)
C4	0.0361 (11)	0.0539 (13)	0.0639 (14)	−0.0057 (9)	0.0147 (10)	0.0025 (11)
C5	0.0343 (11)	0.0526 (12)	0.0543 (13)	0.0006 (9)	0.0143 (9)	−0.0026 (10)
C6	0.0379 (11)	0.0455 (11)	0.0484 (12)	0.0040 (9)	0.0152 (9)	0.0027 (9)
C7	0.0390 (12)	0.0634 (14)	0.0485 (12)	0.0019 (10)	0.0143 (10)	−0.0012 (10)
C8	0.0541 (14)	0.0659 (15)	0.0543 (14)	0.0088 (12)	0.0187 (11)	−0.0031 (12)
C9	0.127 (7)	0.068 (4)	0.079 (5)	0.030 (4)	0.030 (5)	−0.018 (4)
C10	0.186 (10)	0.082 (5)	0.125 (7)	0.023 (6)	0.089 (7)	−0.013 (5)
C9A	0.159 (12)	0.198 (12)	0.192 (12)	0.038 (8)	0.054 (8)	−0.039 (8)
C10A	0.175 (12)	0.194 (13)	0.141 (11)	0.025 (11)	0.041 (10)	0.023 (10)

### Bis[2-amino-5-(ethylsulfanyl)-1,3,4-thiadiazol-3-ium] bis(perchlorato-κO)bis(picolinato-κ2N,O)copper(II) Geometric parameters (Å, º)

**Table:**

Cu1—O1^i^	1.9693 (15)	N1—C5	1.341 (3)
Cu1—O1	1.9693 (15)	N2—H2	0.8600
Cu1—N1	1.9854 (17)	N2—C7	1.307 (3)
Cu1—N1^i^	1.9854 (17)	C1—C2	1.368 (3)
S1—C7	1.731 (2)	C1—C6	1.503 (3)
S1—C8	1.748 (3)	C2—H2A	0.9300
S2—C8	1.730 (3)	C2—C3	1.387 (3)
S2—C9	1.818 (10)	C3—H3	0.9300
S2—C9A	1.93 (2)	C3—C4	1.369 (4)
Cl1—O3	1.428 (2)	C4—H4	0.9300
Cl1—O4	1.408 (2)	C4—C5	1.379 (3)
Cl1—O5	1.421 (2)	C5—H5	0.9300
Cl1—O6	1.444 (2)	C9—H9B	0.9700
O1—C6	1.281 (3)	C9—H9A	0.9700
O2—C6	1.231 (3)	C9—C10	1.394 (12)
N3—N2	1.374 (3)	C10—H10A	0.9600
N3—C8	1.284 (3)	C10—H10B	0.9600
N4—H4A	0.8600	C10—H10C	0.9600
N4—H4B	0.8600	C9A—H9AA	0.9700
N4—C7	1.310 (3)	C9A—H9AB	0.9700
N1—C1	1.344 (3)	C9A—C10A	1.43 (2)
			
O1^i^—Cu1—O1	180.0	C4—C3—H3	120.4
O1—Cu1—N1^i^	97.01 (7)	C3—C4—H4	120.3
O1—Cu1—N1	82.99 (7)	C3—C4—C5	119.5 (2)
O1^i^—Cu1—N1^i^	82.99 (7)	C5—C4—H4	120.3
O1^i^—Cu1—N1	97.01 (7)	N1—C5—C4	121.8 (2)
N1^i^—Cu1—N1	180.0	N1—C5—H5	119.1
C7—S1—C8	87.51 (12)	C4—C5—H5	119.1
C8—S2—C9	99.3 (4)	O1—C6—C1	115.90 (19)
C8—S2—C9A	103.8 (7)	O2—C6—O1	125.3 (2)
O3—Cl1—O6	106.67 (13)	O2—C6—C1	118.8 (2)
O4—Cl1—O3	110.34 (15)	N4—C7—S1	124.9 (2)
O4—Cl1—O5	109.73 (15)	N2—C7—S1	110.12 (18)
O4—Cl1—O6	109.64 (16)	N2—C7—N4	125.0 (2)
O5—Cl1—O3	111.56 (16)	S2—C8—S1	118.33 (16)
O5—Cl1—O6	108.82 (14)	N3—C8—S1	115.4 (2)
C6—O1—Cu1	114.18 (13)	N3—C8—S2	126.3 (2)
C8—N3—N2	109.2 (2)	S2—C9—H9B	107.8
H4A—N4—H4B	120.0	S2—C9—H9A	107.8
C7—N4—H4A	120.0	H9B—C9—H9A	107.1
C7—N4—H4B	120.0	C10—C9—S2	118.0 (9)
C1—N1—Cu1	111.77 (13)	C10—C9—H9B	107.8
C5—N1—Cu1	129.29 (15)	C10—C9—H9A	107.8
C5—N1—C1	118.30 (19)	C9—C10—H10A	109.5
N3—N2—H2	121.1	C9—C10—H10B	109.5
C7—N2—N3	117.7 (2)	C9—C10—H10C	109.5
C7—N2—H2	121.1	H10A—C10—H10B	109.5
N1—C1—C2	122.9 (2)	H10A—C10—H10C	109.5
N1—C1—C6	114.28 (18)	H10B—C10—H10C	109.5
C2—C1—C6	122.8 (2)	S2—C9A—H9AA	113.7
C1—C2—H2A	120.8	S2—C9A—H9AB	113.7
C1—C2—C3	118.4 (2)	H9AA—C9A—H9AB	110.9
C3—C2—H2A	120.8	C10A—C9A—S2	89.9 (15)
C2—C3—H3	120.4	C10A—C9A—H9AA	113.7
C4—C3—C2	119.1 (2)	C10A—C9A—H9AB	113.7
			
Cu1—O1—C6—O2	−179.2 (2)	C2—C3—C4—C5	1.5 (4)
Cu1—O1—C6—C1	−0.1 (2)	C3—C4—C5—N1	−0.2 (4)
Cu1—N1—C1—C2	−170.09 (18)	C5—N1—C1—C2	1.6 (3)
Cu1—N1—C1—C6	10.1 (2)	C5—N1—C1—C6	−178.19 (19)
Cu1—N1—C5—C4	168.70 (18)	C6—C1—C2—C3	179.4 (2)
N3—N2—C7—S1	0.7 (3)	C7—S1—C8—S2	−178.53 (18)
N3—N2—C7—N4	−178.0 (3)	C7—S1—C8—N3	1.2 (2)
N1—C1—C2—C3	−0.3 (4)	C8—S1—C7—N4	177.7 (3)
N1—C1—C6—O1	−6.9 (3)	C8—S1—C7—N2	−1.0 (2)
N1—C1—C6—O2	172.3 (2)	C8—S2—C9—C10	−175.6 (12)
N2—N3—C8—S1	−1.0 (3)	C8—N3—N2—C7	0.2 (3)
N2—N3—C8—S2	178.7 (2)	C9—S2—C8—S1	−176.8 (5)
C1—N1—C5—C4	−1.3 (3)	C9—S2—C8—N3	3.5 (6)
C1—C2—C3—C4	−1.2 (4)	C9A—S2—C8—S1	−158.3 (6)
C2—C1—C6—O1	173.3 (2)	C9A—S2—C8—N3	22.0 (7)
C2—C1—C6—O2	−7.5 (3)		

Symmetry code: (i) −*x*+1, −*y*+1, −*z*.

### Bis[2-amino-5-(ethylsulfanyl)-1,3,4-thiadiazol-3-ium] bis(perchlorato-κO)bis(picolinato-κ2N,O)copper(II) Hydrogen-bond geometry (Å, º)

**Table:**

*D*—H···*A*	*D*—H	H···*A*	*D*···*A*	*D*—H···*A*
N4—H4*A*···O1	0.86	2.14	2.940 (3)	156
N4—H4*A*···O5	0.86	2.59	2.951 (3)	106
N4—H4*B*···O3^ii^	0.86	2.21	2.981 (3)	150
N4—H4*B*···O6^ii^	0.86	2.57	3.005 (3)	113
N2—H2···O2	0.86	1.81	2.656 (3)	170

Symmetry code: (ii) *x*−1, *y*, *z*.
